# The Relationship between Dynapenic Abdominal Obesity and Fall: A Systematic Review and Meta-Analysis of 15,506 Middle to Older Adults

**DOI:** 10.3390/jcm12237253

**Published:** 2023-11-23

**Authors:** Ching-Yun Kao, Yu-Chen Su, Shu-Fang Chang

**Affiliations:** 1 Department of Family Medicine, Saint Paul’s Hospital, Taoyuan 330, Taiwan; cygau0308@gmail.com; 2 Department of Nursing, School of Nursing, National Taipei University of Nursing and Health Sciences, Taipei 112, Taiwan

**Keywords:** dynapenia, abdominal obesity, fall, systematic review, meta-analysis

## Abstract

Background: The main objective of this study was to investigate the risk of falls among middle-aged and older adults with dynapenic abdominal obesity. Methods: A systematic literature search was conducted to review and analyze relevant studies. Dynapenia was measured by handgrip strength, and abdominal obesity was measured by waist circumference. The search keywords included “older people” OR “elderly” OR “middle age” AND “dynapenia” AND “abdominal obesity” AND “fall.” The search was not limited by time and included articles published up until April 2023. The literature search process followed the PRISMA (Preferred Reporting Items for Systematic Reviews and Meta-Analyses) guidelines, involving extraction and examination of the retrieved relevant articles. Systematic literature searches were performed in databases such as Embase, PubMed, MEDLINE, CINAHL, and Cochrane Library. Results: This study collected a total of eight articles with a combined sample size of 15,506 participants. The findings revealed that the average follow-up period for falls was 6.6 years (SD = 3.67). The overall results of the study showed that individuals with dynapenic abdominal obesity had a higher risk of falls compared to those without dynapenic abdominal obesity (RR = 6.91, 95% CI: 5.42–8.80). Subgroup analysis demonstrated that both prospective studies (HR = 6.61; 95% CI = 4.29–10.20) and retrospective studies (OR = 7.37; 95% CI = 5.13–10.59) consistently found a higher risk of falls among individuals with dynapenic abdominal obesity. However, there was no significant difference in fall risk between community-dwelling individuals with dynapenic abdominal obesity and hospitalized individuals with dynapenic abdominal obesity (Q_between_x^2^ = 0.29, *p* = 0.58). Additionally, there was no difference in fall risk between individuals with dynapenic abdominal obesity residing in Europe and Latin America compared to those residing in Asia (Q_between_x^2^ = 0.05, *p* = 0.81). It was worth noting that male individuals with dynapenic abdominal obesity had a higher risk of falls compared to females (Q_between_x^2^ = 4.73, *p* = 0.03). Conclusions: Empirical studies have demonstrated that individuals with dynapenic abdominal obesity have a higher risk of falls. Therefore, healthcare professionals should conduct early fall risk assessments and develop effective preventive strategies specifically targeted at individuals with dynapenic abdominal obesity.

## 1. Background

According to the World Health Organization (WHO) statistics, in 2019, the global population aged 65 and above was approximately 706 million, accounting for 9.0% of the total population. It was projected that by 2050, this percentage will increase to 16.0%, with over 155 million individuals worldwide aged 65 and above. As the global older population continues to grow, geriatric syndromes have become significant health and medical issues [1]. Aging is a natural process characterized by the gradual decline of overall memory and physical abilities. As the body ages and muscle strength decreases, individuals are prone to developing dynapenia, a condition defined as a decline in muscle strength or muscle function, which can adversely affect physical fitness, functional capacity, and overall quality of life in older adults. Dynapenia increases the risk of adverse events such as falls, fractures, and even mortality [2,3].

Studies have shown varying prevalence rates of dynapenia across different countries [4,5,6,7]. In a study conducted in the United Kingdom (UK) and Brazil with 6173 community-dwelling individuals, the prevalence of dynapenia was found to be 7.5% [4]. A study in South Korea with 2652 community-dwelling individuals reported a prevalence of 25.1% [5]. In Japan, a study involving 213 community-dwelling individuals revealed a 10% prevalence of dynapenia [6]. In Colombia, a study with 534 orthopedic outpatients reported a significantly higher prevalence of 84.6% [7]. These findings emphasize the widespread presence of dynapenia among older adults, highlighting the need for attention and proactive measures to mitigate subsequent health issues associated with this condition.

In addition, geriatric abdominal obesity refers to the excessive accumulation of abdominal fat with age, resulting in an increased body fat percentage and structural changes in the body [6]. Geriatric abdominal obesity is a common phenomenon, and its prevalence increases with age. Epidemiological studies [3] conducted globally have shown higher rates of abdominal obesity among older adults compared to other age groups. For example, in the United States, over 60% of adults aged 65 and above are considered to have abdominal obesity. Similarly, in Spain, approximately 32% of men and 39% of women are considered to have abdominal obesity [4]. This highlights that geriatric abdominal obesity is a global health issue with negative implications for the health and quality of life of older individuals [5].

Based on most references [6,8,9,10,11,12,13,14], abdominal obesity is defined by waist circumference: >102 cm for men and >88 cm for women. Dynapenia is defined based on handgrip strength: <26 kg for men and <16 kg for women [6,8,9,10,11,12,13,14]. Research has shown a close association between geriatric abdominal obesity and dynapenia [6]. Abdominal obesity, by increasing body fat percentage, affects muscle metabolism and growth, leading to a decrease in muscle mass and strength. Additionally, adipose tissue in the abdominal region releases harmful cytokines and inflammatory factors that negatively impact muscle health. On the other hand, dynapenia can also contribute to the occurrence and progression of abdominal obesity. As muscles play a crucial role in energy expenditure, a decline in muscle strength leads to a reduced basal metabolic rate, making it easier for fat to accumulate, particularly in the abdominal region [7,15,16]. Furthermore, decreased muscle function diminishes the physical and functional capacity of older adults, further reducing energy expenditure and exacerbating the issue of abdominal obesity. Therefore, the combined effect of dynapenia and geriatric abdominal obesity on falls warrants further investigation. The relationship between these factors and their impact on falls among older adults should be explored.

Falls in older adults are a global health issue. According to a statement from the World Health Organization, people experience disabilities or fatalities due to falls [17]. Among these, individuals aged 65 and older are particularly susceptible to falling. Surveys conducted by the International Fall Prevention Organization indicate that the annual fall rate for individuals aged 65 and above ranges from 30% to 40%, with this proportion increasing with age [16]. Furthermore, more than half of the older population has experienced at least one fall in their lifetime, and approximately 20% of falls result in severe injuries such as fractures, internal bleeding, and even death. Research has shown that dynapenia, which compromises the ability of older adults to support their bodies and maintain balance due to muscle weakness, and abdominal obesity, which alters the body’s center of gravity, increase the risk of falls [17]. The combination of dynapenic abdominal obesity further amplifies the risk of falls among older adults. Previous studies have demonstrated a strong association between dynapenic abdominal obesity and fall [6,8,9,10,11,12,13,14]. However, there is currently a lack of systematic reviews and meta-analyses investigating the differences in fall risk among individuals with dynapenic abdominal obesity across different study populations (hospital vs. community-dwelling), genders (male vs. female), and regions (Brazil, England, and Italy vs. China). Therefore, the primary objective of this study was to examine the association between dynapenic abdominal obesity and the risk of falls in older adults. The secondary objective was to investigate the differences in fall risk among individuals with dynapenic abdominal obesity across different study populations, genders, and country regions. We hypothesize that there are differences in fall risk among individuals with dynapenic abdominal obesity across different study populations, genders, and country regions.

## 2. Methods

This study followed the guidelines of the Preferred Reporting Items for Systematic Reviews and Meta-Analyses (PRISMA) [18].

### 2.1. Data Sources and Search Strategy

The researchers conducted a systematic literature search up to April 2023 on Embase, PubMed, MEDLINE, CINAHL, and Cochrane Library. The search keywords include (older adults OR elderly OR middle age) AND (“dynapenia” AND “abdominal obesity”) AND (fall).

### 2.2. Inclusion and Exclusion Criteria

The eligibility criteria for inclusion in this study were as follows: (1) studies that utilized a prospective or retrospective cohort design; (2) participants were age 60 and above; (3) availability of adjusted or unadjusted odds ratios (ORs) or hazard ratios (HRs) as reported outcomes; (4) inclusion of 95% confidence intervals (CIs); (5) studies published in English with full-text accessibility; and (6) abdominal obesity was defined by waist circumference. Dynapenia was defined by handgrip strength. Exclusion criteria comprised literature review papers, letters to editors, book chapters, postgraduate theses, experimental studies, and master’s and Ph.D. theses.

### 2.3. Data Extraction

Both authors conducted separate examinations and extractions of the researched data, analyzing the study methods, sample sizes, evaluation criteria, and correlations between different stages of depression and frailty. In the event of any inconsistencies observed during data extraction, the authors plan to involve a third reviewer to thoroughly scrutinize the data.

### 2.4. Quality Assessment

The evaluation of cohort studies for selection, comparability, and assessment of outcome/exposure was carried out using the Newcastle–Ottawa Scale (NOS) [19]. The NOS assigns a maximum score of 9, with scores ≥ 7 indicating a low risk of bias, scores of 4–6 indicating a moderate risk of bias, and scores < 4 indicating a high risk of bias. Additionally, the quality of cross-sectional studies was assessed using the Agency for Healthcare Research and Quality (AHRQ) scale. The AHRQ scale was categorized based on the scores: 0–3 indicates low quality, 4–7 indicates medium quality, and 8–11 indicates high quality. Observer reliability was examined by inter- and intra-observer reliability. The kappa values between 0.85 and 0.98, indicating good agreement.

### 2.5. Statistical Analysis

The statistical analysis in this study involved a risk analysis of the relative hazard ratio (HR) and its 95% confidence interval (CI) in examining the association between dynapenic abdominal obesity in older adults and falls. A random-effects model was employed to calculate the overall estimates of HR or odds ratio (OR), assuming that the true underlying effects varied across the studies [14]. Additionally, subgroup comparisons were conducted by stratifying the data based on different populations, gender, and geographical regions. We evaluated the heterogeneity of effect sizes across individual studies by using I^2^ statistics, with proportions greater than 25%, 50%, and 75% considered to have low, moderate, and high heterogeneity, respectively [14]. Data analyses were performed using Comprehensive Meta-Analysis 2.2 (BioStat Solutions, Inc., Englewood, NJ, USA).

## 3. Results

### 3.1. Study Sample

**Figure 1:**
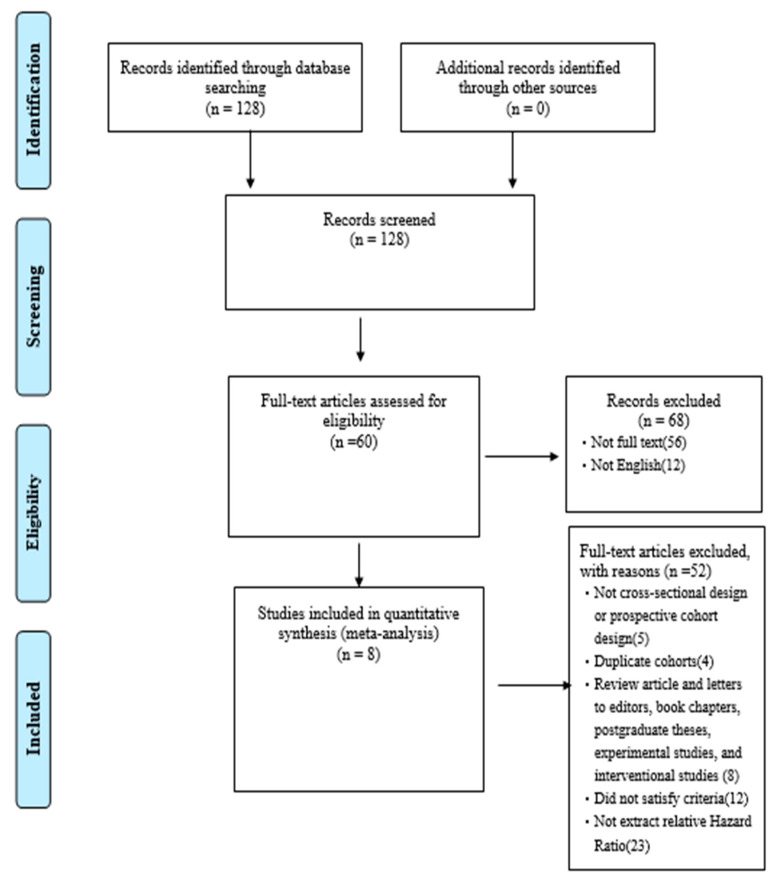
Research flowchart.

This study conducted an analysis of the risk of falls in individuals with dynapenic abdominal obesity based on a total of 15,506 participants across eight studies. A total of four prospective cohort studies and four retrospective studies were included in the analysis. The results indicated that the average follow-up period for falls was 8.75 years (SD = 2.08). The mean BMI and waist circumference of the participants are 32 and 99.7 cm, respectively. The findings of the literature review are illustrated in Figure 1. Initially, several studies were identified; however, some were excluded based on the following criteria: incomplete texts or non-English language, duplicate cohorts, review articles, failure to meet the study criteria, or absence of relevant hazard ratios (HRs) or odds ratios (ORs) extraction. Following the exclusion of such studies, a total of eight cohort studies were selected for inclusion by both reviewers. Table 1 provides a summary of the key characteristics of these eight studies, which were utilized for the meta-analysis.

The final eight studies collectively analyzed 15,506 middle to older adults. As shown in Table 1, four studies adopted a retrospective design, and four were prospective cohort studies. Three studies were from Brazil [8,10,11], three were from England and Italy [9,12,13], and two were conducted in China [6,14]. In addition, there were five studies focused on female studies [6,8,11,13,14], and an additional study targeted males [9]. Meanwhile, there were six studies, including community-dwelling people [8,9,10,11,12,14]. Another person was from the hospital in two studies [6,13].

### 3.2. Quality Assessment

All of the studies scored by NOS indicated a low risk of bias, where the minimum score was eight; the maximum score was nine; and the average score was 8.8 (Table 2).

### 3.3. Association between Dynapenic Abdominal Obesity and Fall (OR or HR)

**Figure 2:**
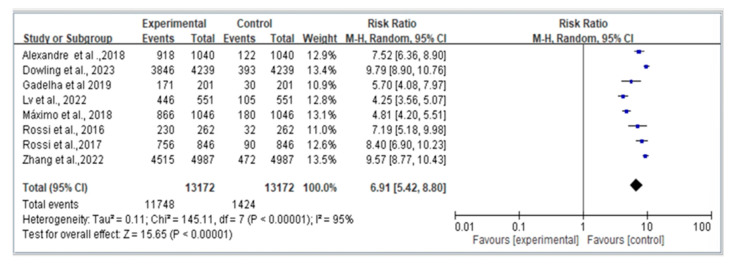
Summary estimates for the dynapenic abdominal obesity and fall outcome [Alexandre et al. [8], Dowling et al. [9], Gadelha et al. [20], Lv et al. [6], Máximo et al. [11], Rossi et al. [12], Rossi et al. [13], Zhang et al. [14]].

Figure 2 presents the results of the meta-analysis conducted on the eight included studies, summarizing the overall effect of dynapenic abdominal obesity on fall risk. The results indicate that individuals with dynapenic abdominal obesity have a significantly higher risk of falls compared to those without dynapenic abdominal obesity (RR = 6.91; 95% CI = 5.42–8.80).

### 3.4. Dynapenic Abdominal Obesity and Fall Outcome (OR)

**Figure 3:**
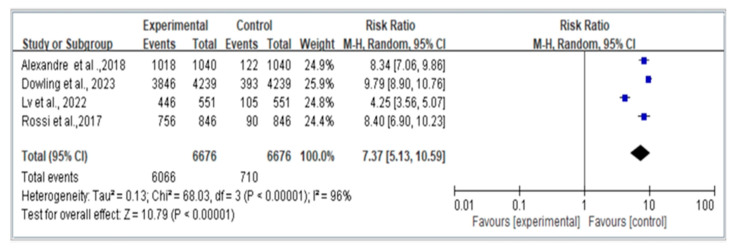
Summary estimates for the dynapenic abdominal obesity and fall outcome (OR) [Alexandre et al. [8], Dowling et al. [9], Lv et al. [6], Rossi et al. [13]].

Figure 3 presents a comparison of four retrospective studies on fall risk using the random-effects model (REM) and displays the odds ratios (OR) and 95% confidence intervals (CI). The results indicate that individuals with dynapenic abdominal obesity have a significantly higher risk of falls compared to those without dynapenic abdominal obesity (OR = 7.37; 95% CI = 5.13–10.59).

### 3.5. Dynapenic Abdominal Obesity and Fall Outcome (HR)

**Figure 4:**
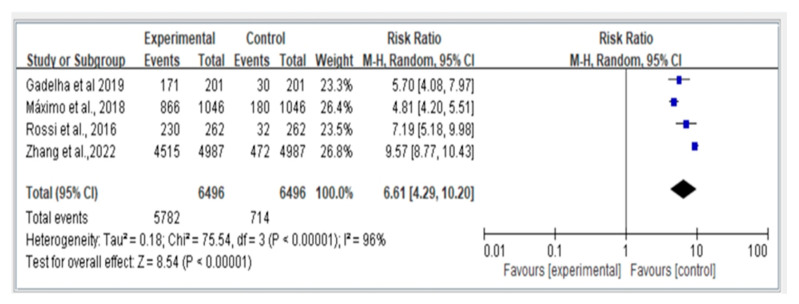
Summary estimates for the dynapenic abdominal obesity and fall outcome (HR) [Gadelha et al. [20], Máximo et al. [11], Rossi et al. [12], Zhang et al. [14]].

Figure 4 depicts a comparison of four prospective studies on fall risk using the random-effects model (REM) and displays the hazard ratios (HR) and 95% confidence intervals (CI). The results indicate that individuals with dynapenic abdominal obesity have a significantly higher risk of falls compared to those without dynapenic abdominal obesity (HR = 6.61; 95% CI = 4.29–10.20).

### 3.6. Subgroup Analysis of Population (Hospital vs. Community-Dwelling)

**Figure 5:**
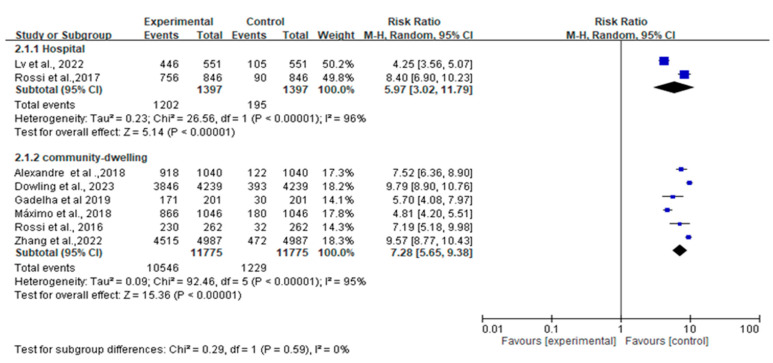
Summary estimates for subgroup the dynapenic abdominal obesity and fall outcome (hospital vs. community-dwelling) [Alexandre et al. [8], Dowling et al. [9], Gadelha et al. [20], Lv et al. [6], de Oliveira Máximo et al. [11], Rossi et al. [12], Rossi et al. [13], Zhang et al. [14]].

Figure 5 presents a comparison of fall risk among different populations, including hospitalized patients and community-dwelling individuals. The results indicate that there is no significant difference in fall risk associated with dynapenic abdominal obesity across different populations (Q_between_x^2^ = 0.29, *p* = 0.58).

### 3.7. Subgroup Analysis of Gender (Male vs. Female)

**Figure 6:**
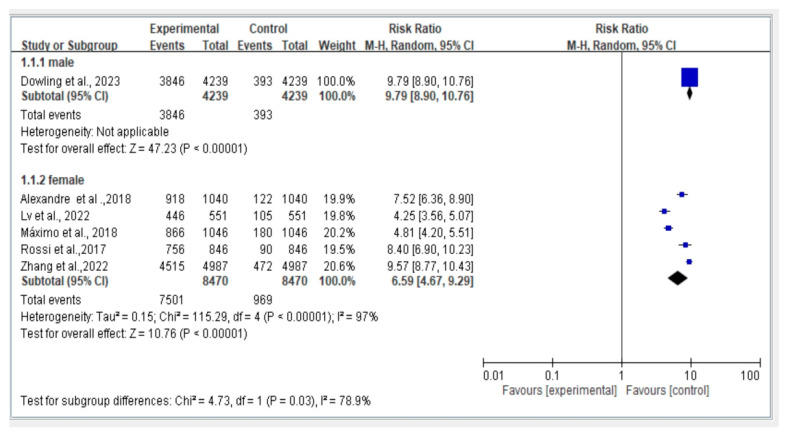
Summary estimates for subgroup the dynapenic abdominal obesity and fall outcome (male vs. female) [Alexandre et al. [8], Dowling et al. [9], Lv et al. [6], Máximo et al. [11], Rossi et al. [13], Zhang et al. [14]].

Figure 6 displays a comparison of fall risk between different genders. Among the included studies, five studies focused on female participants, while one study focused on male participants. Risk ratios were calculated for each gender, and further comparative analysis was conducted using a random-effects model (REM). The results indicate that the fall risk associated with dynapenic abdominal obesity was higher in males compared to females (Q_between_x^2^ = 4.73, *p* = 0.03).

### 3.8. Subgroup Analysis of Regional Area (Europe and Latin vs. Asia)

**Figure 7:**
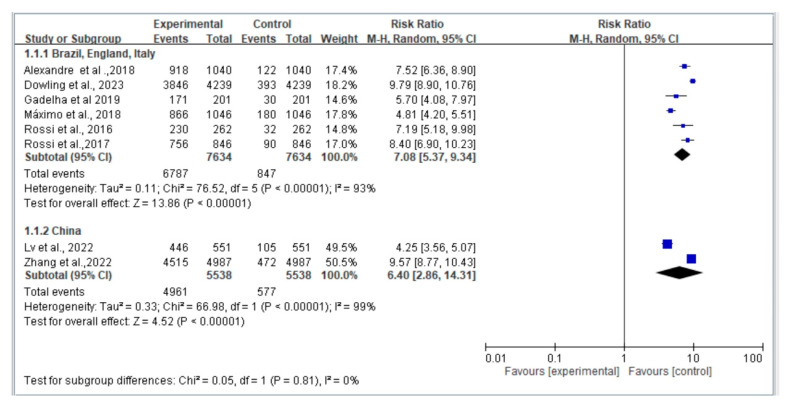
Summary estimates for subgroup the dynapenic abdominal obesity and fall outcome (Brazil, England, Italy vs. China) [Alexandre et al. [8], Dowling et al. [9], Gadelha et al. [20], Lv et al. [6], de Oliveira Máximo et al. [11], Rossi et al. [12], Rossi et al. [13], Zhang et al. [14]].

Figure 7 illustrates a comparison of fall risk stratified by country regions. Among the included studies, six study populations were from Europe and Latin America, while two study populations were from Asia. Risk ratios were calculated for each region, and further analysis was conducted using a random-effects model (REM). The results indicate that there was no significant difference in fall risk associated with dynapenic abdominal obesity among different regions (Q_between_x^2^ = 0.05, *p* = 0.81).

### 3.9. Heterogeneity and Publication Bias

Overall, the between-study heterogeneity determined for dynapenic abdominal obesity and fall comparisons was moderate to high (I^2^ = 95.0%). The results of subgroup analysis showed that the between-study heterogeneity determined for hospital vs. community-dwelling, male vs. female, and Europe and Latin America vs. Asia comparisons was low (I^2^ = 0%), moderate to high (I^2^ = 78.9%), and low (I^2^ = 0.0%), respectively.

## 4. Discussion

The main results of this study confirmed that dynapenic abdominal obesity was a significant factor contributing to fall risk in the middle to older-aged individuals. Anton et al. [21] mentioned the prevention of disability as an important indicator of successful aging, with reducing the risk of falls being a primary goal in preventing disability. Lin et al. [2] and Zhang et al. [14] highlighted that in dynapenic abdominal obesity, older individuals are susceptible to falls, leading to subsequent adverse health consequences such as disability, bed rest, and even mortality, imposing significant burdens on individuals, family caregivers, and society. In 2012, the study showed that direct medical costs amounted to $616.5 million for fatal injuries and $30.3 billion for non-fatal injuries [22]. Furthermore, by 2015, these figures had increased to $637.5 million and $31.3 billion, respectively [22]. Therefore, healthcare professionals must promptly assess the fall risk for dynapenic abdominal obesity in middle-aged and older individuals and provide early care strategies. Researchers [20,23,24,25,26,27,28] pointed out that incorporating exercise regimens that encompass progressive resistance training, along with the implementation of nutritional strategies, such as supplementation with protein and vitamin D, can potentially enhance body composition and muscle function outcomes, ultimately mitigating the risk of falls within this demographic.

Notably, this study conducted an examination based on gender, revealed the concurrent presence of dynapenic abdominal obesity, and demonstrated a correlation with the occurrence of falls in males. de Oliveira Máximo et al. [11] and Dowling et al. [12] investigated gender differences in the relationship between dynapenia and fall risk among older adults. The results showed a stronger correlation between dynapenia and fall risk in males compared to females. Meanwhile, a previous study found that abdominal obesity was associated with an increased risk of falls in older male adults but not in females [10]. Abdominal obesity may compromise balance and postural control in males, thus increasing the likelihood of falls. Therefore, abdominal obesity may have a negative impact on balance and postural control in males, consequently increasing the risk of falls. The existing literature [12] suggests that the coexistence of dynapenia and abdominal obesity in males may increase the risk of falls due to unstable body posture caused by obesity, decreased muscle strength, and impaired neuromuscular function. Further research was needed to explore the underlying mechanisms.

Empirical studies have also shown that there was no significant difference in the risk of falls, whether they are hospitalized patients [6,13] or community-dwelling individuals [8,9,10,11,12,14]. Moreover, the research results indicate that different regions, including England, Italy, Brazil [8,9,10,11,12,13], and China [6,14], show no significant difference in the association between dynapenia and abdominal obesity with falls. This demonstrates that empirical research indicates no significant difference in fall risk between dynapenic individuals with abdominal obesity, whether they are hospitalized, patients, or community residents. Moreover, these findings have been validated across various regions, including Europe, Latin America, and Asia, showing consistent results, and indicating that the fall risk for dynapenic individuals with abdominal obesity does not significantly vary across different geographical areas. These research outcomes contribute to a deeper understanding of factors related to fall risk and provide practical clinical and community care guidelines.

The results indicated that the average follow-up period for falls was 8.75 years (SD = 2.08). However, there was substantial variation in the duration of follow-up across the studies, ranging from a minimum of 1.5 years to a maximum of 9 years. Lin et al. [2] and Gadelha et al. [20] mentioned that the progression of symptoms associated with dynapenic abdominal obesity leading to falls and adverse health events might involve a slow process, necessitating a longer duration of assessment to capture the fall incidence accurately. Nonetheless, such differences in follow-up time may impact the reliability and consistency of the study findings. Shorter follow-up periods may fail to capture potential effects, while longer follow-up durations can provide more extensive long-term information regarding the risk of falls. Thus, the selection of follow-up time was crucial for interpreting and ensuring the reliability of research outcomes, particularly in providing insights into the association between dynapenic abdominal obesity and the risk of falls. Future studies should pay attention to the choice of follow-up time to ensure the accuracy and reliability of research results in further evaluating the impact of dynapenic abdominal obesity on fall risk.

This study has several notable features. Firstly, it was the first systematic review and meta-analysis to examine the relationship between dynapenic abdominal obesity and falls, providing important reference value to the current body of literature. Secondly, the study analyzes the differences in dynapenic abdominal obesity and falls based on different study populations (community or hospital), geographical regions (Brazil, England, and Italy vs. China), and gender (male vs. female), adding novel insights to the existing research. Nonetheless, there are some limitations to this study. Firstly, the included studies had considerable variation in their follow-up durations, ranging from a minimum of 1.5 years to a maximum of 9 years, which may affect the accuracy of estimating fall risks. Secondly, despite controlling for most confounding variables in the majority of studies, our study could not completely address all individual confounders, potentially affecting the overall consistency of the findings. Lastly, due to the limited number of included studies in our meta-analyses, we were unable to visually assess funnel plots for potential publication bias. However, despite these limitations that may have influenced the conclusions and inferences drawn from our meta-analysis, we believe that the findings of this study are valuable for healthcare professionals and can serve as a basis for further development of care strategies for individuals with dynapenic abdominal obesity.

### 4.1. Future Research Directions

This study found that only a few studies had compared the risk of dynapenic abdominal obesity and falls with different genders. Therefore, it is worthy of further analysis in the future. In addition, this study found that the criteria for determining dynapenia in various studies are not consistent, and currently, no consensus has been reached. It is worthwhile to convene an international consensus meeting to discuss specific and consistent cut points in dynapenic abdominal obesity to provide important guidelines for medical care.

### 4.2. The Practical Applications

This study found that dynapenic abdominal obesity is highly correlated with falls. In particular, male adults with dynapenic abdominal obesity had a higher risk of falling than females. Therefore, healthcare providers should pay special attention to older males with dynapenic abdominal obesity. Healthcare professionals have highlighted the significance of integrating exercise programs that include progressive resistance training, coupled with the adoption of nutritional interventions such as protein and vitamin D supplementation. This integration holds the potential to optimize body composition and muscle function outcomes, ultimately reducing the vulnerability to falls among male individuals.

## 5. Conclusions

Dynapenic abdominal obesity is an important issue in current older adults’ care. Empirical research has shown an association between dynapenic abdominal obesity and adverse health events related to falls, with consistent results across different population attributes and geographical regions. Notably, male individuals with dynapenic abdominal obesity exhibit a higher risk of falling compared to their female counterparts. Therefore, healthcare professionals must promptly assess the risk of falls and provide effective intervention strategies for this high-risk group to mitigate the subsequent risks of fractures and disabilities.

## Figures and Tables

**Table 1:** Characteristics of the included studies for meta-analysis between dynapenic-abdominal obesity and fall.

No.	First Author (Year)	Population	Sample Size	Country	Sex	Age	Length of Follow-up	OR (95% CI)	HR (95% CI)	Variable Adjusted
1	Alexandre et al. [8]	Community-dwelling	3374	Brazil	F	50	4–8 years	2.10 (0.01–0.03)	None	socioeconomic, behavioral, clinical characteristics, and BMI
2	Dowling et al. [9]	Community-dwelling	4239	England	M	60–87	2 years	2.10 (1.30–3.20)	None	age and sex
3	Gadelha et al. [20]	Community-dwelling	201	Brazil	Both	60–80	1.5 years	None	3.60 (1.32–9.82)	None
4	Lv et al. [6]	Hospital	551	China	F	65	5 years	3.39 (1.47–7.81)	None	age, sex, marital status, education, and BMI
5	de Oliveira Máximo [11]	Community-dwelling	1046	Brazil	F	60	8 years	None	2.06 (1.04–4.10)	sex, age, floor with different levels, polypharmacy, body mass index, diabetes, joint disease, dizziness/vertigo, depressive symptoms, and functional status
6	Rossi et al. [12]	Community-dwelling	262	Italy	Both	66–78	5.5 years	None	3.39 (1.91–6.02)	age, gender
7	Rossi et al. [13]	Hospital	846	Italy	F	65–95	9 years	2.10 (1.14–3.88)	None	age, sex, smoking habit, education, medications, and diabetes
8	Zhang et al. [14]	Community-dwelling	4987	China	F	60	14 years	None	1.28 (1.02–1.60)	age, sex, and BMI

Note: BMI (Body Mass Index) = kg/m^2^.

**Table 2:** The results of Newcastle–Ottawa scale quality assessment for cohort studies.

First Author (Year)	Selection				Comparability	Outcome			
	Representativeness of the Exposed Cohort	Selection of the Non-Exposed Cohort	Ascertainment of Exposure	Demonstration That Outcome of Interest Was Not Present at Start of Study	Comparability of Cohorts on the Basis of the Design or Analysis	Assessment of Outcome	Was Follow-Up Long Enough for Outcomes to Occur?	Adequacy of Follow-Up of Cohorts	Overall Quality Score (Maximum = 9)
Alexandre et al. [8]	★	★	★	★	★	★	★	★	**8**
Dowling et al. [9]	★	-	★	★	★	★	★	★	**7**
Gadelha et al. [20]	★	★	★	★	★	★	★	★	**8**
Lv et al. [6]	★	★	★	★	★	★	★	★	**8**
de Oliveira Máximo et al. [11]	★	★	★	★	★	★	★	★	**8**
Rossi et al. [12]	★	-	★	★	★	★	★	★	**7**
Rossi et al. [13]	★	★	★	★	★	★	★	★	**8**
Zhang et al. [14]	★	-	★	★	★	★	★	★	**7**

Note: Yes = ★; No = -

## Data Availability

Data are available upon request due to privacy/ethical restrictions.
